# Beneficial Effects of a Protein Rich Diet on Coping Neurotrans-mitter Levels During Ampicillin-Induced Neurotoxicity Compared to Propionic-Acid Induced Autistic Biochemical Features

**Published:** 2016-08-09

**Authors:** Ramesa Shafi Bhat, Kaushal Kishore Chandrul, Afaf El-Ansary

**Affiliations:** 1 *Department of Biochemistry, Science College, King Saud University, Riyadh, Saudi Arabia.*; 2 *Department of Pharmaceutical Science, Shri Venkateshwara University, Gajraula, Amroha, Uttar Pradesh, India.*; 3 *Central Laboratory, Female Center for Medical Studies and Scientific Section, King Saud University, Riyadh, Saudi Arabia.*

**Keywords:** Ampicillin, propionic acid, neurotransmitter, brain

## Abstract

This study examined the effects of a protein rich diet on coping neurotransmitter levels in orally administered ampicillin–induced neurotoxic rats compared with propionic acid (PA) models of autism. 40 young male western albino rats were divided into four groups. The first group served as control and received phosphate buffered saline orally; the second group serving as autistic model was treated with oral dose of PA (250 mg/kg body weight/day for 3 days); the third group was treated with the neurotoxic dose of ampicillin (50 mg/kg for three weeks); the fourth group received the same dose of ampicillin and was fed with special protein rich diets. Noradrenaline, dopamine, serotonin glutamate, glutamine and interleukin 6 (IL-6) were measured in the brain homogenate of all tested groups. Specified doses of PA and ampicillin significantly (P<0.001) decreased noradrenaline, dopamine, and serotonin levels when compared to control. Also glutamate, IL-6 levels were significantly (P<0.001) increased in PA treated group while non-significant increase was found in ampicillin treated group. Non-significant increase of glutamine was found in PA treated group with a significant increase in ampicillin treated group. The effects of ampicillin on these parameters were found to be potentiated when the rats were fed on a protein rich diet. Our results end with the conclusion that dietary protein level may be a useful tool to find out a path to restrict neurotransmitter alterations in neurodevelopmental disorders like autism.

*Autism spectrum disorder (ASD) is a lifelong neurodevelopmental disorder characterized by impaired social communication and a pattern of rigid and repetitive behavior and restricted interests (* 1 *). *Several studies have suggested that children with ASD have a history of increased antibiotic use for recurrent infections prior to their diagnosis (2) and other studies have suggested that antibiotic use during pregnancy is linked to the development of ASD (3). Some groups have even hypothesized that use of specific antibiotics early in life could be causative for ASD (4) and others have suggested that antibiotic use early in life facilitates a vicious cycle between immune system impairment and dysbiosis (5) Antibiotics can also disturb normal flora to allow the overgrowth of *Clostridium difficile,* which in turn has been associated with the development of autism (6) These bacteria are spore-formers capable of resisting antibacterial drugs. If the antibiotics are discontinued, the spores germinate and produce toxins and metabolites, including short-chain propionic acid (PA), which has recently been reported to induce persistent biochemical and behavioral autistic features in rat pups (7-9). Ampicillin (Amp) is believed to exert an inhibitory effect on gamma-aminobutyric acid (GABA) transmission due to its beta-lactam ring structure, which is somewhat similar to the GABA structure (10). Imbalance in GABAergic/ glutamat-ergic, serotonergic, dopaminergic neurotransmiss-ion together with neuroinflammation were recently recorded as the most important signals that are impaired and related to clinical presentation and severity of autism (11).

Neurotransmitters play important role in normal growth and development of the brain. The levels of neurotransmitters tend to be skewed in individuals within the autism spectrum. Various studies strongly suggest that neurochemical factors could play a major role in autism. As such, pharmaceutical treatments for autism focus on modulating neurotransmitter levels known to play a role in the symptoms of autism including serotonin, dopamine and noradrenaline. Increasing numbers of studies are showing that daily supplements of proteins often effectively reduce patients’ symptoms, because they are directly converted into neurotransmitters. The effects of high-protein diets have been of great interest in the last decade. Supplementation with high-protein diets is often used to improve physical status causing an effective reduction in body weight, fat deposition and improving plasma lipid profile (12). Some studies have shown the beneficial effects of high-protein diets on rodent brain such as protecting against cerebral ischemia and reducing apoptosis in the ischemic cortex (13, 14). However, little is known regarding the effects of high-protein diet and autism in the presence of antibiotics. The development of animal models of autism is one approach that could help identify the mechanism by which autism develops in humans. Thus, rodent model with autistic features was developed through orally administered neurotoxic dose of PA (15) and Amp (16) and effect of high protein diet in shrinking the neurotoxic effect was analyzed by measuring neurotransmitters.

## Materials and methods

**Experimental animals**

The experimental assays for this study were performed on 40 young (approximately 21 days old) male western albino rats (45 to 60 g). Rats were obtained from animal house at the pharmacy college in King Saud University and allowed to drink water ab libitum for a period of one week before stating the treatment.

**Experimental design**

Animals were randomly assigned to four groups of ten rats each. The first group of rats (n=10) received only phosphate buffered saline and were used as a control group. The second group was given oral neurotoxic doses of PA (250 mg/kg body weight/day for three days) (17) and were referred to as the oral buffered PA-treated group. The third group received an orogastric dose of ampicillin (50 mg/ kg for three weeks) with standard diet and referred to as the ampicillin group (16). Animals of the last group were given orogastric doses of ampicillin (50 mg/kg for three weeks), and were fed with a high-protein diet for 10 weeks. The Ethics Committee at King Saud University approved the protocol of the present test, in addition, all experiments were performed in accordance with the guidelines of the National Animal Care and Use Committee.

**Diets**

The control protein diet and the protein enriched diets (corresponding to the amount of casein present) were prepared according to the protocol of the Institutional Animal care and Use committee (18). The diets composition has been shown in Table 1.

**Tissue preparation**

At the end of the feeding trial, the rats were anesthetized with carbon dioxide and decapitated. The brain was removed from the skull and was dissected into small pieces and homogenized as a whole in 10 times w/v bi-distilled water. Selected samples were kept at−80 °C until further use 

**Assay of neurotransmitters (noradrenaline, dopamine, serotonin)**

The concentrations of noradrenaline, dopamine, serotonin were determined in brain homogenates using high-performance liquid chromatography with electrochemical detection (HPLC-ED) (19). Brain tissue was homogenized in 150 µl 0.1 M perchloric acid containing 0.4 mM sodium metabisulphite using ultrasonic cell disrupter. The homogenates were then centrifuged at 10,000 g at 4 °C for 25 min and the supernatants were filtered through a 0.22 μm filter (Sigma) and frozen at -70 °C until analysis. Filtrate was injected into the HPLC system which consisted of a quaternary gradient delivery pump (HP 1050, Hewlett-Packard), a sample injector (Model 7125, Rheodyne, Berkeley), and an analytical column (ODS 2 C18, 4.6 x 250 mm, Hewlett-Packard) protected by a guard column (Lichnospher 100 RP-18, 4 X 4 mm), particle size 5 µm (Hewlett-Packard). The mobile phase comprised a 0.15 M sodium dihydrogen phosphate, 0.1 mM EDTA, 0.5 mM sodium octanesulphonic acid, 10-12% methanol (v/v) and 5 mM lithium chloride. The mobile phase was adjusted to pH 3.4 with phosphoric acid, filtered through 0.22 μm filter (Sigma) and degassed with helium. A column temperature of 32 ^o^C and a flow rate of 1.4 ml/min was used.The electrochemical detector (HP 1049 A, Hewlett-Packard) with glassy carbon working electrode was used at a voltage setting of +0.65 V for monoamines. The detector response was plotted and measured using a chromatointegrator. The concentration of noradrenaline, dopamine, and serotonin in each sample was calculated from the integrated chromatographic peak area and expressed as ng/100 mg wet tissue.

**Assay of IL-6**

IL-6 was assayed using a Quantikine ELISA kit (R & D Systems, Minneapolis, MN, USA). A microplate was precoated with a monoclonal antibody specific for rat IL-6. 50 μL of each standard, control, or sample were placed in separate wells. The reagent was mixed by gently tapping the plate frame for 1 min after being covered with the adhesive strip provided. The plate was incubated for 2 h at room temperature and the immobilized antibody bound any rat IL-6 present.

After washing away unbound substances, an enzyme linked polyclonal antibody specific for rat IL-6 was added to the wells. Following a subsequent wash step to remove unbound antibody- enzyme reagents, 100 μL of substrate solution was added to each well and the plate was incubated for30 min at room temperature. The enzymatic reaction yielded a blue product that turned yellow when the stop solution was added. The intensity measured for the color, was in proportion to the amount of rat IL-6 bound in the initial step. The sample values were then read off the standard curve.

**Table 1:** Composition of the experimental diets

**Diet**	**Protein (%)**	**Casein (%)**	**Starch (%)**	**Fat (%)**	**Salt mixture (%)**	**Vitamin mixture (%)**	**Ash (%)**
Control	20	10	65	5	4	2	4
Protein enriched	40	25	30	5	4	2	4

**Glutamine and glutamate analysis**

Rat brain glutamine (Gln) or glutamate (Glut) were measured independently using ELISA kit, a product of Cusabio. Antibody specific for Gln, or Glut has been pre-coated onto a microplate. Standards and samples were pipetted into the wells where the respective immobilized antibody could bind any Gln or Glut present. After removing any unbound substances, a biotin-conjugated antibody specific for Gln or Glut was added to the wells. After washing, avidin conjugated horseradish peroxidase (HRP) was added to the wells. Following a wash to remove any unbound avidin-enzyme reagent, a substrate solution was added to the wells and color developed in proportion to the amount of Gln or Glut bound in the initial step. The color development was then stopped and the intensity of the color was measured. The minimum detectable dose was typically less than 19.5 pmol/ml and 3.12 nmol/ml for Gln and Glut, respectively.

**Statistical analysis**

The data were analyzed using the Statistical Package for the Social Sciences (SPSS, Chicago, IL, USA). The results were expressed as mean± standard deviation of the mean (SD). All statistical comparisons between the control, PA and Amp-treated rat groups were performed using the one-way analysis of variance (ANOVA) test complemented with the Dunnett test for multiple comparisons. Significance was assigned at the level of p<0.05. Receiver operating characteristics curve (ROC) analysis was performed. Area under the curve (AUC), cutoff values, and degree of specificity and sensitivity were calculated. Pearson’s correlations were performed between the measured parameters.

## Results

Table 2 shows the percentage change in addition with mean± S.D of noradrenaline, dopamine, serotonin, IL-6, Gln and Glut in the brain homogenates of the four groups of rats. There was a significant depletion in the noradrenaline (67.51% and 85.42%), dopamine (85.42% and 85.42%), serotonin (85.42% and 85.42%) levels in the brain of the PA and Amp treated rat respectively, compared to the control groups (P<0.001). However, feeding with high protein diet for 10 weeks post antibiotic treatment in group IV significantly reversed the noradrenaline (92.61%), dopamine (105.94%), serotonin (93.05%) back to normal levels compared to control. IL-6, Gln and Glut were elevated in all groups compared to that of control. IL6 and Glut activity was significantly higher in PA-treated group (130.07% and 156.30%, respectively P< 0.001), while non-significant in Amp (106.07% and 156.30%, respectively). Protein diet with post antibiotic treatment reversed the IL6 and Glut activity values near to control group. On the other hand, Gln levels were increased in all treated groups as compared to control (Table 2).

**Fig 1:**
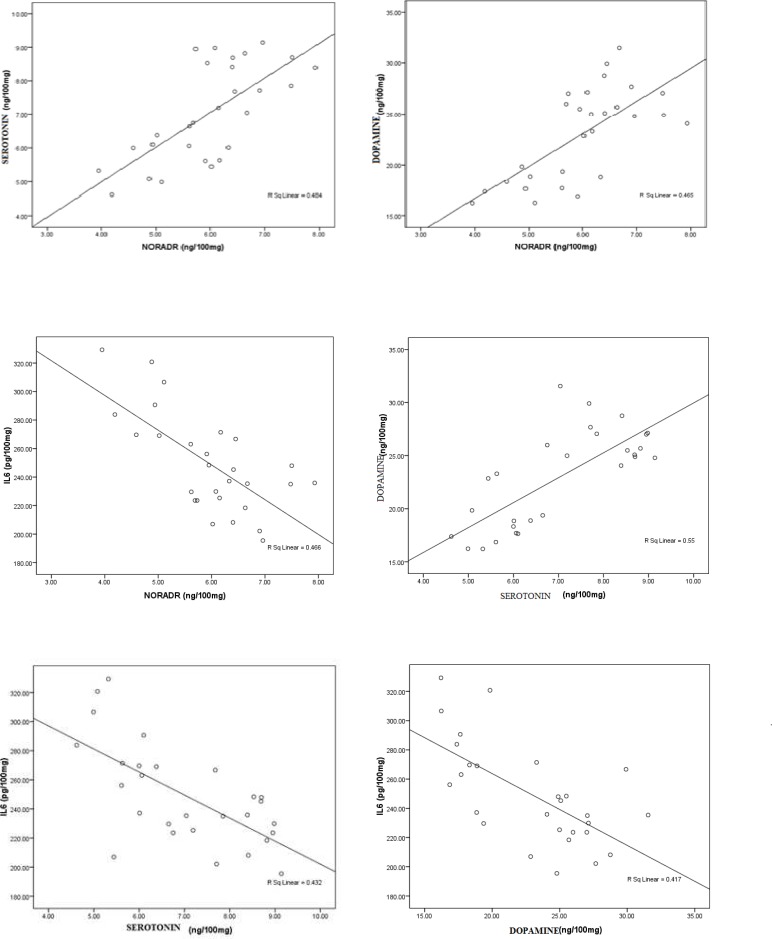
Correlation between A: noradrenaline (ng/100mg) and serotonin (ng/100mg) (positive correlation); B: noradrenaline (ng/100mg) and dopamine (ng/100mg) with best fit line curve (positive correlation); C: noradrenaline (ng/100mg) and IL6 (pg/100mg) with best fit line curve (negative correlation); D: serotonin (ng/100mg) and dopamine (ng/100mg) with best fit line curve (positive correlation); E: serotonin (ng/100mg) and IL6 (pg/100mg) with best fit line curve (negative correlation); F: dopamine (ng/100mg) and IL6 (pg/100mg) with best fit line curve (negative correlation

Table 3 and Figure 1 present Pearson’s correlations between the measured parameters. There was a significant positive correlation (Pearson's R=0.696; P=0.001) between noradrenaline~serotonin (Pearson's R=0.682; P=0.001) between noradrenaline ~dopamine, (Pearson's R=0.742; P=0.001) between serotonin ~dopamine and between Glut/Gln~ Gln (R=0,593; P=0.001). There was also a significant negative correlation between noradrenaline~IL6 (R=-0.683; P=0.001), serotonin~IL6 (R= 0.657; P= 0.001) and dopamine~IL6 (R=0.646; P=0.001) Glut/Gln ~ Glut (R=-0,599; P= 0.001).

**Fig 2:**
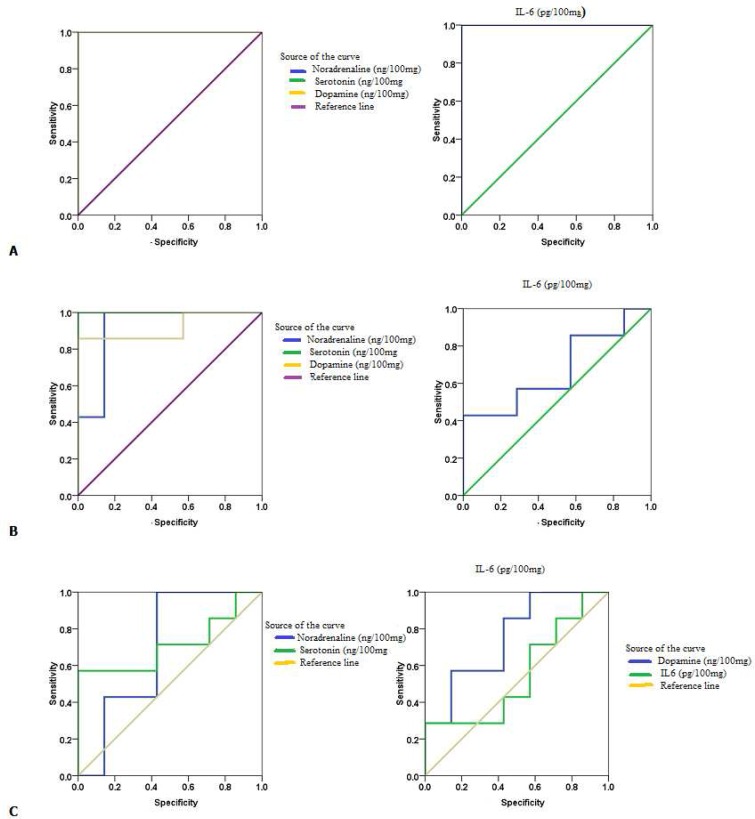
ROC curve of all parameters in A: PA group; B: ampicillin group; C: protein group

Receiver operating characteristics curves are collectively presented in figure 2 as curve A, B and C. Area under the curve (AUC), cutoff values, sensitivity and specificity are listed in Table 4.

Table 5 demonstrates the multiple regression analysis using noradrenaline, dopamine, and serotonin, and IL6, Gln and Glut as dependent variables.

**Table 2:** Biochemical analyzes in the four studied groups

**Parameter**	**Group**	**N**	**Min.**	**Max.**	**Mean ± S.D.**	**Percent** **Change**	**P ** **value ** a	**P ** **value ** b
**Noradrenaline, (ng/100mg)**	Control	10	5.73	7.93	6.92 ± 0.78	100.00		0.001
	Propionic acid	10	3.95	5.11	4.67 ± 0.45	67.51	0.001	
	Ampicillin	10	5.61	6.33	5.91 ± 0.28	85.42	0.013	
	Protein	10	5.95	6.90	6.40 ± 0.35	92.61	0.150	
**Serotonin, (ng/100mg)**	Control	10	7.85	9.14	8.59 ± 0.42	100.00		0.001
	Propionic acid	10	4.62	6.38	5.50 ± 0.66	64.01	0.001	
	Ampicillin	10	5.44	6.75	6.02 ± 0.51	70.10	0.001	
	Protein	10	7.04	8.98	7.99 ± 0.78	93.05	0.109	
**Dopamine, (ng/100mg)**	Control	10	24.06	28.75	25.95 ± 1.68	100.00		0.001
	Propionic acid	10	16.21	19.84	17.79 ± 1.34	85.42	0.001	
	Ampicillin	10	16.86	25.99	20.70 ± 3.37	85.42	0.005	
	Protein	10	24.99	31.55	27.49 ± 2.45	105.94	0.195	
**IL6 (pg/100mg)**	Control	10	195.48	247.96	227.34± 19.47	100.00		0.001
	Propionic acid	10	269.06	329.31	295.71± 23.92	130.07	0.001	
	Ampicillin	10	206.98	271.41	241.15± 23.26	106.07	0.252	
	Protein	10	202.16	266.70	232.31± 20.84	102.19	0.653	
**Glutamate (pmol/ml)**	Control	7	215.20	265.83	237.99± 18.93	100.00		0.011
	Propionic acid	7	269.47	553.81	371.97± 105.29	156.30	0.001	
	Ampcillin	7	223.79	373.43	294.03± 57.76	123.55	0.252	
	Protein	7	208.63	332.02	269.17± 49.42	113.10	0.194	
**Glutamine** **(pmol/ml)**	Control	7	1866.05	2502.77	2169.73± 223.94	100.00		0.001
	Propionic acid	7	2178.22	3982.39	2930.67± 664.56	135.07	0.023	
	Ampcillin	7	2340.84	3767.09	2921.69± 520.35	134.66	0.008	
	Protein	7	2861.86	3716.80	3212.59± 347.65	148.06	0.001	
**Glutamate/ Glutamine** **(pmol/ml)**	Control	7	7.87	11.19	9.15 ± 1.11	100.00		0.042
	Propionic acid	7	4.69	12.52	8.36 ± 2.80	91.34	0.001	
	Ampcillin	7	6.78	12.82	10.15 ± 2.02	110.87	0.252	
Protein	7	8.62	17.82	12.34 ± 3.08	134.81	0.026

a : P value between control group and each other group using independent samples t-Test;

b : P value between all groups using one-way ANOVA.

**Table 3:** Pearson’s correlations between the measured biochemical parameters

**Parameters**	**R(Person correlation)**	**Sig.**	
Noradrenaline (ng/100mg) ~ serotonin, (ng/100mg)	0.696**	0.001	P
Noradrenaline (ng/100mg) ~ dopamine (ng/100mg)	0.682**	0.001	P
Noradrenaline (ng/100mg) ~ IL6 (pg/100mg)	-0.683**	0.001	N
Serotonin, (ng/100mg) ~ dopamine (ng/100mg)	0.742**	0.001	P
Serotonin, (ng/100mg) ~ IL6 (pg/100mg)	-0.657**	0.001	N
Dopamine (ng/100mg) ~ IL6 (pg/100mg)	-0.646**	0.001	N
Glutamate /Glutamine ~ Glutamate	-0.599**	0.001	N
Glutamate /Glutamine ~ Glutamine	0.593**	0.001	P

**: correlation is significant at the 0.01 level; P: positive correlation; N: negative correlation.

**Table 4:** ROC-Curve of biochemical parameters in all groups

**Parameter**	**Group**	**Area under the curve**	**Cut-off value**	**Sensitivity %**	**Specificity %**
Noradrenaline (ng/100mg)	Propionic acid	1.000	5.420	100.0 %	100.0 %
	Ampicillin	0.918	6.365	100.0 %	85.7 %
	Protein	0.694	6.930	100.0 %	57.1 %
Serotonin, (ng/100mg)	Propionic acid	1.000	7.115	100.0 %	100.0 %
	Ampicillin	1.000	7.300	100.0 %	100.0 %
	Protein	0.714	7.780	57.1 %	100.0 %
Dopamine (ng/100mg)	Propionic acid	1.000	21.950	100.0 %	100.0 %
	Ampicillin	0.918	23.675	85.7 %	100.0 %
	Protein	0.755	24.940	100.0 %	42.9 %
IL6 (pg/100mg)	Propionic acid	1.000	258.510	100.0 %	100.0 %
	Ampicillin	0.673	252.070	42.9 %	100.0 %
	Protein	0.551	248.145	28.6 %	100.0 %
Glutamate (pmol/ml)	Propionic acid	1.000	267.650	100.0 %	100.0 %
	Ampicillin	0.878	257.650	85.7 %	85.7 %
	Protein	0.667	249.605	66.7 %	71.4 %
Glut-amine (pmol/ml)	Propionic acid	0.918	2551.090	71.4 %	100.0 %
	Ampicillin	0.959	2517.300	85.7 %	100.0 %
	Protein	1.000	2682.315	100.0 %	100.0 %
Glutamate/ Glutamine (pmol/ml)	Propionic acid	0.612	7.436	42.9 %	100.0 %
	Ampicillin	0.694	9.185	71.4 %	71.4 %
Protein	0.881	10.297	83.3 %	85.7 %

**Table 5:** Multiple regression using stepwise method for biochemical parameters as dependent variables

Parameter	Predictor Variable	Beta	P value	Adjusted R square	Model	
					F value	P value
Noradrenaline (ng/100mg)	Serotonin (ng/100mg)	0.469	0.001	0.464	24.399	0.001
	serotonin (ng/100mg)	0.293	0.019	0.540	16.837	0.001
	IL6 (pg/100mg)	-0.011	0.030			
Serotonin (ng/100mg)	Dopamine (ng/100mg)	0.234	0.001	0.533	31.791	0.001
	Dopamine (ng/100mg)	0.158	0.007	0.587	20.177	0.001
	Noradrenaline (ng/100mg)	0.526	0.046			
Dopamine (ng/100mg)	Serotonin (ng/100mg)	2.347	0.001	0.533	31.791	0.001
IL6 (pg/100mg)	Noradrenaline (ng/100mg)	-24.384	0.001	0.446	22.734	0.001
Glutamate (pmol/ml)	Glutamate/ Glutamine	-1.075	0.001	0.896	117.714	0.001
	Glutamate	0.887	0.001			
Glutamate/ Glutamine (pmol/ml)	Glutamine	-0.020		0.333	13.967	0.001
	Glutamine	-0.025	0.001	0.920	156.263	0.001
Glutamate	0.003	0.001

## Discussion

In the present study, a significant decrease in noradrenaline, dopamine, serotonin content with increased levels of Glut and Gln was shown in the brain of the PA model of autism. Our study evaluated the role of protein diet in preventing the neurochemical alterations in brain caused by exposure to Amp (Table 2). Our findings revealed that protein diet attenuates some of the neurochemical changes that are induced by Amp exposure.

Compared with control group, PA and Amp-treated rats demonstrated lower noradrenaline, dopamine, serotonin levels. Abnormalities in neurotransmitter systems have frequently been reported in PA administrated rodent models of autism (20, 21). PA has recently been reported to induce persistent biochemical and behavioral autistic features in rat pups, and Amp has proven to promote overgrowth of propionicbacteria *Klebsiella pneumonia* which in turn can induce neurotoxicity. The results of the present experiments demonstrated that Amp treatment for three weeks has affected the neurotransmitter levels in brain of rats which was almost the same as that found in PA model of autism. Amp administration has previously been shown to disturb microbiome and promote the overgrowth of propionobacteria; hence can be connected with development of autism in our animal model. Amp treatment along with protein rich diet induced satisfactory improvement of neurotransmitter levels in brain tissue. The synthesis of neurotransmitters in mammalian brain responds rapidly to changes in precursor availa-bility. All neurotransmitters are made from amino acids except acetylcholine. Serotonin synthesis depends largely on the brain concentra-tions of L-tryptophan, its precursor amino acid. Also, the synthesis of catecholamines (e.g., dopa-mine, norepinephrine) in the brain varies with the availability of the precursor amino acid L-tyrosine. Protein diet induced changes in blood amino acid concentrations, and as a result, will influence the synthesis of neurotransmitters in the brain.

IL-6 is normally expressed at relatively low levels in the brain (22, 23). However, elevated cytokine response is associated with autism and IL-6 has been repeatedly found to be increased in the autistic brain (24, 25). Wei et al. (26) developed a mouse model overexpressing IL-6 in the brain with an adenoviral gene delivery approach and confirmed that IL-6 is an important mediator of autism-like behaviors. We found a significant increase in IL-6 levels in PA-treated group (30.07%) when compared with normal controls. However, Amp treatment did not seem to have a major impact on IL6 levels in brain tissue. These results can be supported by the report that Amp is able to decrease the blood level of IL-6 by inhibiting prostaglandin E2 synthesis (27). Furthermore, it was found that Amp treatment with protein rich diet was able to shrink the IL-6 levels auxiliary; which can be supported by the recent finding that proteins rich diets can reduce the IL-6 levels in blood (28). The significant increase of brain Glut in PA and Amp treated groups can easily be related to ASD features as Glut excitotoxicity is one of the most important mechanisms involved in the etiology of autism.

In addition to the AUC, the specificity and sensitivity values listed in figure 2 and Table 4 demonstrate the possibility of using noradrenaline, dopamine, serotonin and IL-6 as markers of PA and Amp neurotoxicity. All measured parameters demonstrated satisfactory sensitivity and very high specificity, which confirmed that PA and Amp can excite toxicity and neuroinflammation.

## Conflict of interest

The authors declared no conflict of interest.
